# The Usefulness of Inflammation-based Prognostic Scores for the Prediction of Postoperative Mortality in Patients Who Underwent Intestinal Resection for Acute Intestinal Ischemia

**DOI:** 10.7759/cureus.6372

**Published:** 2019-12-13

**Authors:** Veli Vural, Omer vefik Ozozan

**Affiliations:** 1 General Surgery, Akdeniz University Hospital, Antalya, TUR; 2 General Surgery, Istinye University, Istanbul, TUR

**Keywords:** mesenteric ischemia, serum marker, mortality

## Abstract

Objective

The current study was conducted to clarify whether the neutrophil-lymphocyte ratio (NLR) and platelet-lymphocyte ratio (PLR) are clinically useful in predicting postoperative mortality among patients undergoing surgery for acute intestinal ischemia (AII).

Materials and methods

The study was conducted as a retrospective investigation of 37 consecutive patients operated for AII between January 2014 and September 2019. Data regarding potential prognostic factors, including age, sex, preoperative white blood cell count (WBC), C-reactive protein (CRP), neutrophil, lymphocyte, and platelet counts were obtained from medical records.

Results

Univariate analysis revealed that age, WBC, and neutrophil count were predictors of postoperative mortality. In multivariate analysis, age (OR =1.14; 95% CI, 1.005-1.303; P=0.02) was found to be the only independent variable predicting postoperative mortality.

Conclusions

Preoperative NLR and PLR cannot be used as independent variables to predict postoperative 30-day mortality in patients with AII who underwent surgery.

## Introduction

Acute intestinal ischemia (AII) has a prevalence of up to one in 1,000 hospital admissions. Rapid diagnosis is imperative because the clinical consequences can be mortal (67% to 80%) [1-2]. AII may be caused by a number of mechanisms such as acute thromboembolism, extrinsic compression, or nonocclusive mesenteric ischemia [2]. The definitive diagnosis of intestinal ischemia is difficult and often delayed. Clinical signs such as peritonitis develop later in the clinical course, and it usually indicates full-thickness infarction in the intestinal wall [3]. In this situation, intestinal resection is the only option for treatment.

Despite the clinical severity and relatively high prevalence of AII, factors predicting postoperative mortality are poorly defined. There are many clinical variables proposed by researchers to predict postoperative mortality after intestinal resection such as advanced age, male sex, and emergency surgery, but there is no clear consensus regarding major risk factors [4].

We hypothesized that mortality after intestinal resection may be associated with an impaired immune response of the patients. The aim of this study was to evaluate the prognostic value of inflammation-based prognostic scores, including the neutrophil-lymphocyte ratio (NLR) and platelet-lymphocyte ratio (PLR) in patients who underwent intestinal resection for AII.

## Materials and methods

Patient data

Between January 2014 and September 2019, 37 consecutive patients had intestinal resection for AII in our institute. A retrospective review was conducted on patient files. The following data were collected: age, sex, and preoperative laboratory findings, including white blood cell count (WBC), platelet count (PLT), neutrophil count, lymphocyte count, and C-reactive protein (CRP) level. NLR and PLR were calculated for each patient. The primary outcome criteria were defined as 30-day mortality.

Statistical analysis

The Statistical Package for the Social Sciences v16.0 (SPSS Inc., Chicago, Illinois, USA) for Windows was used for statistical analyses of the data. The Shapiro-Wilk test was used for assessing normality. All values are expressed as mean ± standard deviation or counts (percentage) unless otherwise specified. For a comparison of the two groups, the Mann-Whitney U test was used to further evaluate clinical variables in study groups. Multivariate logistic regression (LR) analysis with backward feature selection was performed to evaluate the independent factors associated with mortality. Variables with p < 0.1 at univariate analysis were included in a final multivariate model. In the multivariate logistic regression analyses, the results were presented as an odds ratio (OR) with a 95% confidence interval. We measured the performance of variables using receiver operating characteristic (ROC) curves and calculated positive likelihood ratios for cut-point with high sensitivity or high specificity. The discrimination of a marker is considered perfect if the area under the curve (AUC) is equal to one, good if AUC is greater than 0.8, moderate if AUC is 0.6-0.8, and poor if AUC is less than 0.6. A p-value lower than 0.05 was considered statistically significant.

## Results

There were 19 (51.4 %) male patients and 18 (48.6 %) female patients. The mean age of the patients was 67.8±16.8 (20-99) years. Nine patients died within 30 days after surgical operation, resulting in a mortality rate of 24.3%. Univariate analysis showed three variables associated with a higher risk of postoperative mortality: age (p=0.044), WBC (p=0.025), and preoperative neutrophil count (p=0.032) (Table 1). Age (OR =1.14; 95% CI, 1.005-1.303; p=0.02) was the only variable significantly associated with postoperative mortality in the multivariate analysis. The area under the ROC curve of the age for predicting postoperative mortality was 0.737 (95% CI, 0.576-0.897; p=0.044) and the cut-off was 72 years old.

**Table 1:** Clinical characteristics of the patients WBC: White Blood Cell, PLT: Platelet, NEU: Neutrophil, LYM: Lymphocyte, NLR: Neutrophil-to-Lymphocyte Ratio, PLR: Platelet-to-Lymphocyte Ratio, CRP: C-Reactive Protein

		No mortality			Mortality			p
		Mean	Standard Deviation	Count (%)	Mean	Standard Deviation	Count (%)	
Age		64.0	17.1		78.6	9.3		0.044
Sex	Male			15 (78.9%)			4 (21.1%)	p>0.05
	Female			13 (72.2%)			5 (27.8%)	p>0.05
WBC (per mm3)		11923.6	6795.1		19601.1	10189.3		0.025
PLT (per mm3)		282714.3	139114.1		311777.8	217475.2		p>0.05
NEU (per mm3)		9876.8	6305.9		16704.4	8977.9		0.032
LYM (per mm3)		1057.9	557.4		1421.1	923.9		p>0.05
NLR		11.9	11.6		16.0	9.9		p>0.05
PLR		364.4	292.3		314.6	252.9		p>0.05
CRP (mg/L)		9.4429	8.5244		13.4891	9.1067		p>0.05

## Discussion

In this study, we investigated the utility of inflammation-based prognostic scores as independent risk factors for postoperative mortality in patients with acute intestinal ischemia who underwent intestinal resection. We found that age was independently associated with postoperative mortality and age higher than 72 years was predictive for mortality.

An accurate prediction of outcome after an emergency surgery would better assist clinicians when discussing mortality with the patients and their families and guiding difficult treatment decisions [5]. The physiological immune response of circulating leucocytes to various stressful events is often characterized by an increase in neutrophil count and a decline in lymphocyte count along with a decrease in platelet count [6]. The NLR and PLR are simple, readily available laboratory variables. Due to the fact that they are continuous variables, NLR and PLR are also able to reflect acute changes in the inflammatory state of a patient, which may lead to more accurate and dynamic results [6-10]. NLR has been suggested as potentially useful, yet few studies have considered the role of NLR in predicting perioperative outcomes [8]. Some studies have shown that in certain conditions (i.e., chronic renal failure, autoimmune diseases, and cardiovascular diseases), a better predictor of inflammation is the PLR, outlining a superior performance for PLR over NLR [6]. We observed no significant difference for the NLR and PLR between patients with and without mortality.

Emergency surgery forms a significant part of the general surgeon’s workload. The significant associations between age and postoperative mortality are unsurprising and consistent with previous research [4-5,11-12]. A recent retrospective review showed that 8% of patients will undergo a surgical procedure in their final week of life [13]. The postoperative mortality rate after emergency surgery is higher in old people, even for conditions usually regarded as having low mortality [5,11]. A previous study reported the overall morbidity (26%) and mortality (22%) in emergency abdominal surgery of patients >65 years of age. Mortality increases with every decade of age beyond 50 years, reaching 40% to 50% in those aged 80 years and above [11]. Predicting mortality in the older population can be difficult. Although age is a predictor of mortality, the presence of co-morbidities is likely a stronger predictor of poor outcomes [11-12]. Several studies have tried to combine a few risk factors to predict outcomes. Among these combinations are the American Society of Anesthesiologists (ASA) fitness grade and the nature of the procedure (elective, urgent, or emergency) [12,14]. Duron et al. demonstrated that postoperative mortality after major digestive surgery increased significantly among patients aged 65-74 years, and age ≥65 years was by itself an independent risk factor for mortality (odds ratio (OR), 2.21; 95% (CI), 1.36-3.59; p=0.001). Also, six independent risk factors of postoperative mortality were characteristic of elderly patients: age ≥85 years, emergency surgery, anemia, WBC > 10,000/mm³, ASA class IV, and a palliative cancer operation [15]. In brief, emergency surgery should be centralized to improve outcomes in old patients.

The limitations of our study are its retrospective design and the low number of patients. Further, well-designed prospective studies are required. There were also problems with documentation.

## Conclusions

In conclusion, preoperative NLR and PLR cannot be used as independent variables for predicting postoperative mortality in patients with acute intestinal ischemia after intestinal resection.

## References

[1] Kairaluoma MI, Karkola P, Heikkinen E, Huttunen R, Mokka REM, Larmi TKI (1977). Mesenteric infarction. Am J Surg.

[2] Martin B (2007). Prevention of gastrointestinal complications in the critically ill patient. AACN Adv Crit Care.

[3] Woo K, Major K, Kohanzadeh S, Allins AD (2007). Laparotomy for visceral ischemia and gangrene. Am Surg.

[4] Acosta-Merida MA, Marchena-Gomez J, Hemmersbach-Miller M, Roque-Castellano C, Hernandez-Romero JM (2006). Identification of risk factors for perioperative mortality in acute mesenteric ischemia. World J Surg.

[5] Kettunen J, Paajanen H, Kostiainen S (1995). Emergency abdominal surgery in the elderly. Hepato Gastroenterol.

[6] Kotfis K, Ślozowska J, Safranow K, Szylińska A, Listewnik M (2019). The practical use of white cell inflammatory biomarkers in prediction of postoperative delirium after cardiac surgery. Brain Sci.

[7] Chiarelli M, Achilli P, Tagliabue F (2019). Perioperative lymphocytopenia predicts mortality and severe complications after intestinal surgery. Ann Transl Med.

[8] Vaughan-Shaw P, Rees JRE, King AT (2012). Neutrophil lymphocyte ratio in outcome prediction after emergency abdominal surgery in the elderly. Int J Surg.

[9] de Jager CP, van Wijk PT, Mathoera RB, de Jongh-Leuvenink J, van der Poll T, Wever PC (2010). Lymphocytopenia and neutrophil-lymphocyte count ratio predict bacteremia better than conventional infection markers in an emergency care unit. Crit Care.

[10] Jiang N, Deng JY, Liu Y, Ke B, Liu HG, Liang H (2014). The role of preoperative neutrophil-lymphocyte and platelet-lymphocyte ratio in patients after radical resection for gastric cancer. Biomarkers.

[11] Desserud KF, Veen T, Soreide K (2016). Emergency general surgery in the geriatric patient. Br J Surg.

[12] Duron JJ, Duron E, Dugue T (2011). Risk factors for mortality in major digestive surgery in the elderly: a multicenter prospective study. Ann Surg.

[13] Kwok AC, Semel ME, Lipsitz SR, Bader AM, Barnato A, Gawande AA, Jha AK (2011). The intensity and variation of surgical care at the end of life: a retrospective cohort study. Lancet.

[14] Byrne BE, Bassett M, Rogers CA, Anderson ID, Beckingham I, Blazeby JM (2018). Short-term outcomes after emergency surgery for complicated peptic ulcer disease from the UK National Emergency Laparotomy Audit: a cohort study. BMJ Open.

[15] Novello M, Mandarino FV, Di Saverio S (2019). Post-operative outcomes and predictors of mortality after colorectal cancer surgery in the very elderly patients. Heliyon.

